# Geometric sieve over number fields for higher moments

**DOI:** 10.1007/s40993-023-00466-6

**Published:** 2023-08-02

**Authors:** Giacomo Micheli, Severin Schraven, Simran Tinani, Violetta Weger

**Affiliations:** 1 Department of Mathematics, University of South Florida, 4202 E. Fowler Avenue, Tampa, FL 33620 USA; 2 Department of Mathematics, University of British Columbia, 1984 Mathematics Road, Vancouver, BC V6T 1Z2 Canada; 3 Institute of Mathematics, University of Zurich, Winterthurerstrasse 190, 8057 Zurich, Switzerland; 4 Department of Electrical and Computer Engineering, Technical University of Munich, Theresienstrasse 90, 80333 Munich, Germany

**Keywords:** Densities, Number Fields, Expected Values, Variance

## Abstract

The geometric sieve for densities is a very convenient tool proposed by Poonen and Stoll (and independently by Ekedahl) to compute the density of a given subset of the integers. In this paper we provide an effective criterion to find all higher moments of the density (e.g. the mean, the variance) of a subset of a finite dimensional free module over the ring of algebraic integers of a number field. More precisely, we provide a geometric sieve that allows the computation of all higher moments corresponding to the density, over a general number field *K*. This work advances the understanding of geometric sieve for density computations in two ways: on one hand, it extends a result of Bright, Browning and Loughran, where they provide the geometric sieve for densities over number fields; on the other hand, it extends the recent result on a geometric sieve for expected values over the integers to both the ring of algebraic integers and to moments higher than the expected value. To show how effective and applicable our method is, we compute the density, mean and variance of Eisenstein polynomials and shifted Eisenstein polynomials over number fields. This extends (and fully covers) results in the literature that were obtained with ad-hoc methods.

## Introduction

For a positive integer *d*, the density of a set $T \subseteq \mathbb {Z}^d$ is defined as

$$\begin{aligned} \rho (T) = \lim \limits _{H \rightarrow \infty } \frac{ \mid T \cap [-H,H[^d \mid }{ (2H)^d}, \end{aligned}$$

if it exists. Computing the density of a subset *T* of ${\mathbb {Z}}^n$ is a classical problem in number theory, as it provides an estimate on the relative size of *T* and $\mathbb Z^n$.

A well-known result in this area is the density of coprime pairs, which has been computed by Mertens [15] and Césaro [6, 7] independently. This result has been generalized to coprime *m*-tuples by Nymann [21] and further to rectangular unimodular matrices by Micheli and Weger [19]. Another interesting target set is the set of Eisenstein polynomials, where the results are due to Dubickas [8] in the case of monic polynomials and due to Heymann and Shparlinski [11] in the non-monic case. In addition, Micheli and Schnyder computed the density of shifted and affine Eisenstein polynomials over ${\mathbb {Z}}$ in [17]. Since the density can also be defined over a general number field, further generalizations of these results appeared (see [10, 19]), using a pull-back function from the set of interest, i.e., the ring of algebraic integers to the integers.

An elegant tool to compute certain densities over the integers is called the geometric sieve, or local to global principle. This principle was introduced by Poonen and Stoll [22] (independently also by Ekedahl [9]) and uses a local characterization of the target set, i.e., over the *p*-adic integers. In fact, all of the above mentioned examples have a density that can be computed through this tool. The geometric sieve has been generalized to number fields by Bright et al. in [3] and to function fields by Micheli in [16]. Other interesting works for geometric sieves include [2, 4, 23, 25].

Recently, a new question regarding densities has arisen in [14]: to compute the expected value and the variance of Eisenstein polynomials over the integers. More precisely, the question can be formulated as *“On an average, for a random Eisenstein polynomial how many primes p are such that this Eisenstein polynomial satisfies the criterion of Eisenstein for p?”* This led to [18], where a general definition of the expected value corresponding to the density is given and an addendum to the geometric sieve over the integers is provided, which allows to compute these expected values corresponding to the density directly using the local characterization of the target set. In addition, in the thesis [26] the variance of Eisenstein polynomials and other target sets over the integers were determined using an adaption of this new geometric sieve. In [12] the second author et al. generalized the geometric sieve for higher moments over function fields.

This paper finally closes all the missing gaps in this historical overview, as we provide a final extension of the geometric sieve over number fields, which allows to compute all higher moments of a target set. As an application of this result, we compute the missing density of Eisenstein polynomials over number fields, in addition to their mean and variance.

The paper is organized as follows: in Sect. 2 we recall the definition of density over the integers as well as over number fields and restate the original geometric sieve by Poonen and Stoll and its generalization to number fields by Bright et al. In Sect. 3 we give the definition of the expected value and higher moments of a system and present the main result, Theorem 3.5, an extension of the geometric sieve, which works over a general number field and allows to compute any higher moment. Finally, we compute the density, the mean and the variance of Eisenstein polynomials over number fields in Sect. 4.

## Preliminaries

For a positive integer *d*, the density of a set $T \subseteq \mathbb {Z}^d$ is defined by restricting to a *d*-dimensional cube of height *H* and letting *H* go to infinity.

### Definition 2.1

(Density) Let $d \in \mathbb {N}.$ The density of $T \subseteq \mathbb {Z}^d$ is defined to be

$$\begin{aligned} \rho (T) = \lim \limits _{H \rightarrow \infty } \frac{ \mid T \cap [-H,H[^d \mid }{ (2H)^d}, \end{aligned}$$

if the limit exists.

Similarly, one can define the upper density $\overline{\rho }$ and the lower density $\underline{\rho }$ using the limit superior and the limit inferior, respectively.

There exist various tools to compute the density of a set, one of the main tools is the geometric sieve [22, 24] by Poonen and Stoll, which allows to compute the density of certain sets by characterizing these sets over the *p*-adic integers.

To present the principle we need to introduce the following notation. For a set *S* we denote by $2^S$ its powerset and by $S^C$ its complement. In addition, if *S* is a subset of a metric space we denote by $\partial (S)$ its boundary. We denote by $\mathcal {P}$ the natural primes and for $p \in \mathcal {P}$ we denote by $\mathbb {Z}_p$ the *p*-adic integers. Let $M_{\mathbb {Q}} = \{ \infty \} \cup \mathcal {P}$ be the set of all places of $\mathbb {Q},$ where we denote by $\infty$ the unique Archimedean place of $\mathbb {Q}.$ Finally, for $d \in \mathbb {N},$ we denote by $\mu _\infty$ the Lebesgue measure on $\mathbb {R}^d$ and by $\mu _p$ the normalized Haar measure on $\mathbb {Z}_p^d.$

### Theorem 2.2

[[24, Lemma 1]] Let *d* be a positive integer. Let $U_{\infty } \subseteq \mathbb {R}^d$, such that $\mathbb {R}_{\ge 0} \cdot U_{\infty } = U_{\infty }$ and $\mu _{\infty }(\partial (U_{\infty }))=0.$ Let $s_{\infty }= \frac{1}{2^d}\mu _{\infty }(U_{\infty } \cap [-1,1]^d)$. For each prime *p*, let $U_p \subseteq \mathbb {Z}_p^d$ be such that $\mu _p(\partial (U_p)) =0$ and define $s_p = \mu _p(U_p)$. Define the following map

$$\begin{aligned} P: \mathbb {Z}^d\rightarrow & {} 2^{M_{\mathbb {Q}}}, \\ a\mapsto & {} \left\{ \nu \in M_{\mathbb {Q}} \mid a \in U_{\nu } \right\} . \end{aligned}$$

If the following is satisfied:

1 $$\begin{aligned} \lim _{M \rightarrow \infty } \bar{\rho }\left( \left\{ a \in \mathbb {Z}^d \mid a \in U_p \ \text {for some prime} \ p > M \right\} \right) =0, \end{aligned}$$

then:

i) $$\sum \limits _{\nu \in M_{\mathbb {Q}}} s_{\nu }$$ converges.
ii) For $\mathcal {S} \subseteq 2^{M_{\mathbb {Q}}},$ $\rho (P^{-1}(\mathcal {S}))$ exists, and defines a measure on $2^{M_{\mathbb {Q}}}$.
iii) For each finite set $S \in 2^{M_{\mathbb {Q}}}$, we have that
  $$\begin{aligned} \rho (P^{-1}(\{S\})) = \prod _{\nu \in S} s_{\nu } \prod _{\nu \not \in S} (1-s_{\nu }), \end{aligned}$$
  and if $\mathcal {S}$ consists of infinite subsets of $2^{M_{\mathbb {Q}}}$, then $\rho (P^{-1}(\mathcal {S}))=0.$

To show that Condition (1) is satisfied, one can often apply the following lemma.

### Lemma 2.3

[22, Lemma 2] Let *d* and *M* be positive integers. Let $f,g \in \mathbb {Z}[x_1, \ldots , x_d]$ be relatively prime and define

$$\begin{aligned} S_M(f,g) = \{ a \in \mathbb {Z}^d \mid f(a) \equiv g(a) \equiv 0 \mod p \ \text {for some prime} \ p >M\}. \end{aligned}$$

Then

$$\begin{aligned} \lim \limits _{M \rightarrow \infty } \overline{\rho }(S_M(f,g)) =0. \end{aligned}$$

The notion of density, as well as the geometric sieve can be generalized to number fields. For this we will first recall some basics.

Let *K* be a number field and denote by $\mathfrak {o}$ its ring of integers. For any non-zero prime ideal $\mathfrak {p}$ of $\mathfrak {o}$ we denote the completion of *K* with respect to the non-Archimedean place $\mathfrak {p}$ by $K_\mathfrak p$. Furthermore, we denote by $\mathfrak {o}_\mathfrak {p}$ the ring of integers of $K_\mathfrak p$. Note that $\mathfrak {o}_\mathfrak {p}$ is compact in the subspace topology of $K_\mathfrak p$. Hence, there exists a unique normalized Haar measure on $\mathfrak {o}_\mathfrak {p}$, which we denote by $\nu _\mathfrak {p}$. We define the degree of the number field *K* to be the index $k=[K: \mathbb {Q}]$, which is finite by assumption. Thus, one has that $\mathfrak {o}$ is isomorphic to $\mathbb {Z}^k$ as a $\mathbb {Z}$-module. Let $\mathbb {E}=\{ e_1, \dots , e_k \}$ be an integral basis for $\mathfrak {o}$, then we can define for $H\in \mathbb {N}$ the following set

$$\begin{aligned} O(H,\mathbb {E}) = \left\{ \sum _{j=1}^k a_j e_j \in \mathfrak {o}\, \ a_j \in [-H, H[ \cap \mathbb {Z} \right\} . \end{aligned}$$

Let $d\in \mathbb {N}$ and $T \subseteq \mathfrak {o}^d$, then we define the upper, respectively the lower density on the number field *K* as

$$\begin{aligned} \overline{\rho }_\mathbb {E}(T) = \limsup _{H\rightarrow \infty } \frac{\vert T \cap O(H, \mathbb {E})^d \vert }{(2H)^{dk}} \end{aligned}$$

and

$$\begin{aligned} \underline{\rho }_\mathbb {E}(T) = \liminf _{H\rightarrow \infty } \frac{\vert T \cap O(H, \mathbb {E})^d \vert }{(2H)^{dk}}, \end{aligned}$$

respectively. If $\overline{\rho }_\mathbb {E}(T) = \underline{\rho }_\mathbb {E}(T)$, we also define the density of *T* with respect to the basis $\mathbb {E}$ as

$$\begin{aligned} \rho _\mathbb {E}(T) = \lim _{H\rightarrow \infty } \frac{\vert T \cap O(H, \mathbb {E})^d \vert }{(2H)^{dk}}. \end{aligned}$$

Furthermore, in the case where $\rho _\mathbb {E}(T)$ is independent of the integral basis $\mathbb {E}$, we simply write $\rho (T)$. For a non-zero prime ideal $\mathfrak {p} \subset \mathfrak {o}$, we say that $\mathfrak {p}$ is lying above *p* for some $p \in \mathcal {P}$, if $\mathfrak {p} \cap \mathbb {Z}= p \mathbb {Z}$ and we write $\mathfrak {p} \vert p$. Finally, we denote by $\mathcal {P}_K$ all non-zero prime ideals in $\mathfrak {o}$.

In [3], Bright et al. provide a generalization of the original geometric sieve to number fields. We will state the result translated to our setting. Let us define the $\mathbb {R}$-algebra $k_\infty =\mathfrak {o}\otimes _\mathbb {Z} \mathbb {R}$, which has covolume $|\Delta _K|^{1/2}$, where $\Delta _K$ denotes the discriminant of *K* and $k_\infty$ is endowed with the Haar measure $\nu _{\infty }$, see [20, Proposition I.5.2]. That is, for some set $X \subseteq k_\infty$ we have

$$\begin{aligned} \nu _\infty (X)= \frac{1}{|\Delta _K |^{1/2} } \, \mu _\infty (f(X)), \end{aligned}$$

where $\mu _\infty$ denotes the Lebesgue measure and *f* is the isomorphism $K \otimes _\mathbb {Q} \mathbb {R} \rightarrow \mathbb {R}^{k}$ from [20, Proposition I.5.1]. Let us denote $\mathcal {P}_K \cup \{ \infty \}$ by $N_K$.

### Theorem 2.4

[3, Proposition 3.2] Let *K* be a number field and $k = [K: \mathbb {Q}].$ Let $\mathbb {E}$ be an integral basis of $\mathfrak {o}$ and let *d* be a positive integer.

For each $\mathfrak {p} \in \mathcal {P}_K$, let $U_\mathfrak {p} \subseteq \mathfrak {o}_\mathfrak {p}^d$ be such that $\nu _\mathfrak {p}(\partial ( U_\mathfrak {p})) = 0$ and define $s_\mathfrak {p}= \nu _\mathfrak {p}(U_\mathfrak {p}).$ Let $U_\infty \subseteq k_\infty ^d$, such that $\nu _\infty (\partial (U_\infty ))=0$ and $\mathbb {R}_{\ge 0} \cdot U_\infty = U_\infty$. We define $s_\infty = \frac{1}{2^{dk}}\nu _\infty \left( U_\infty \cap \left( [-1,1] \cdot O(1, \mathbb {E})^d\right) \right)$ Define the following map

$$\begin{aligned} P: \mathfrak {o}^d&\rightarrow 2^{N_K}, \\ a&\mapsto \{ \eta \in N_K \ \mid \ a \in U_\eta \}. \end{aligned}$$

If the following is satisfied:

2 $$\begin{aligned} \lim _{M \rightarrow \infty } \overline{\rho }_\mathbb {E} \left( \mathfrak {o}^d \cap \bigcup _{p>M} \bigcup _{\mathfrak {p}\in \mathcal {P}_K, \ \mathfrak {p} \vert p} U_\mathfrak {p} \right) = 0, \end{aligned}$$

then:

1. $$\sum \limits _{\eta \in N_K} s_\eta $$ converges.
2. For $\mathcal {S}\subseteq 2^{N_K}$, $m(\mathcal {S}) = \rho _\mathbb {E}(P^{-1}(\mathcal {S}))$ exists and defines a measure on $2^{N_K}$.
3. The measure *m* is concentrated at the finite subsets of $N_K$. For each finite set $S \in 2^{N_K}$, we have that
  3 $$\begin{aligned} m(\{ S\}) = \prod _{\eta \in S} s_\eta \prod _{\eta \notin S} (1-s_\eta ) \end{aligned}$$
  and if $\mathcal {S}$ consists of infinite subsets of $2^{N_K}$ then $m(\mathcal {S})=0$.

### Remark 2.5

Note that for any two integral bases $\mathbb {E}_1, \mathbb {E}_2$ there exists an integer *c* such that for all $H\in \mathbb {N}$ holds $O(H,\mathbb {E}_1) \subseteq O(cH, \mathbb {E}_2)$, and thus if (2) holds for one integral basis, then it holds for all integral bases.

In addition, we have a similar result to Lemma 2.3 over number fields, in order to show that Condition (2) is satisfied. For *M* a positive integer and $\mathfrak {p} \in \mathcal {P}_K$, consider the unique prime integer $p \in \mathcal {P}$ with $\mathfrak {p} \mid p$. We write $\mathfrak {p} \succ M$ if and only if $p>M$. Similarly one defines $\mathfrak {p} \preceq M$. In addition, we will assume that $\infty \preceq M$ for all $M \in \mathbb {N}$.

### Lemma 2.6

Let *d* and *M* be positive integers. Let $f,g \in \mathfrak {o}[x_1, \ldots , x_d]$ be relatively prime. Define

$$\begin{aligned} S_M(f,g) = \left\{ a \in \mathfrak {o}^d \mid f(a) \equiv g(a) \equiv 0 \mod \mathfrak {p} \ \text {for some prime ideal} \ \mathfrak {p} \succ M \right\} , \end{aligned}$$

then we have for every integral basis $\mathbb {E}$ of $\mathfrak {o}$

$$\begin{aligned} \lim _{M \rightarrow \infty } \bar{\rho }_\mathbb {E}(S_M(f,g)) = 0. \end{aligned}$$

### Proof

This follows directly from [3, Lemma 3.3] applied to the subscheme defined by $f=0=g$. $\square$

## Higher moments

In [18] the authors generalized Theorem 2.2, the geometric sieve, also called local to global principle, over the integers, to expected values. We will now generalize Theorem 2.4, the geometric sieve over number fields, to higher moments.

Before we introduce the definition of expected values of systems $(U_\eta )_{\eta \in N_K}$, let us notice that for a general number field *K*, $\eta =\infty$ does no longer correspond to an Archimedean place. Still we find it useful to include this possiblity to modifity the box $O(H, \mathbb {E})^d$. Even though $N_K$ does no longer correspond to the set of all places, we will keep using the same notation in order to stay consistent with our previous paper [18]. Note that the set of elements living in infinitely many $U_\eta$, i.e.,

$$\begin{aligned} I= \{ A \in \mathfrak {o}^d \mid A \in U_\eta \ \text {for infinitely many} \ \eta \in N_K\} \end{aligned}$$

has density zero; this follows directly from Condition (2). Let us denote by $O(H, \mathbb {E})_I = O(H, \mathbb {E}) {\setminus } I.$

### Definition 3.1

Let *d* be a positive integer and assume that $U_\eta$ satisfies the assumptions of Theorem 2.4 for all $\eta \in N_K$, then we define *the expected value of the system* $( U_\eta )_{\eta \in N_K}$ to be

$$\begin{aligned} \mu _\mathbb {E} = \lim \limits _{H \rightarrow \infty } \displaystyle {\frac{\sum \limits _{A \in O(H, \mathbb {E})_I^d } |\{ \eta \in N_K \mid A \in U_{\eta } \} |}{ (2H)^{kd}}}, \end{aligned}$$

if it exists. More generally, for any non-negative integer *n* we define *the n-th moment of the system* $(U_\eta )_{\eta \in N_K}$ to be

$$\begin{aligned} \mu _{n,\mathbb {E}} = \lim \limits _{H \rightarrow \infty } \displaystyle {\frac{\sum \limits _{A \in O(H,\mathbb {E})_I^d } |\{ \eta \in N_K \mid A \in U_{\eta } \}|^n }{ (2H)^{kd}} }, \end{aligned}$$

if it exists.

This limit essentially gives the expected value of the number of $\eta$, such that a random element in $\mathfrak {o}^d$ is in $U_\eta$.

### Definition 3.2

For a set $T\subseteq \mathfrak {o}^d$, we say that a *system* $(U_\eta )_{\eta \in N_K}$ *corresponds to T*, if Condition (2) is satisfied and $T^C= P^{-1}(\{\emptyset \})$.

As in [18] we can restrict Definition 3.1 to subsets of $\mathfrak {o}^d$, i.e., we define the *n*-th moment of the system $(U_\eta )_{\eta \in N_K}$ restricted to $T \subseteq \mathfrak {o}^d$ to be

$$\begin{aligned} \mu _{n,T, \mathbb {E}} = \lim \limits _{H \rightarrow \infty } \displaystyle {\frac{\sum \limits _{A \in O(H, \mathbb {E})_I^d\cap T } |\{ \eta \in N_K \mid A \in U_{\eta } \} |^n }{ |O(H, \mathbb {E})_I^d \cap T |} }, \end{aligned}$$

if it exists. We will write $\mu _{T,\mathbb {E}}$ for $\mu _{1,T, \mathbb {E}}$ and if it does not depend on the integral basis $\mathbb {E}$, we will just write $\mu _{n,T}$, respectively $\mu _T$ for $\mu _{1,T}$. Note, that this is similar to the conditional expected value.

For any non-negative integer *n*, one can easily pass from $\mu _{n,\mathbb {E}}$ to $\mu _{n,T, \mathbb {E}}$ and vice versa. The proof is the same as in [18, Lemma 11].

### Lemma 3.3

If the density of *T* with respect to $\mathbb {E}$ exists and is non-zero and *T* is such that $T^C \subseteq P^{-1}(\{ \emptyset \})$, then $\mu _{n,T, \mathbb {E}}$ exists if and only if $\mu _{n, \mathbb {E}}$ exist. In addition, we have that $\mu _{n, \mathbb {E}}=\mu _{n,T, \mathbb {E}} \cdot \rho _\mathbb {E}(T)$.

We have the following straightforward corollary from Theorem 2.4.

### Corollary 3.4

Let $\eta _1, \dots , \eta _n \in N_K$ with $\eta _i \ne \eta _j$ for $i\ne j$ and let $U_{\eta _j}$ be chosen as in Theorem 2.4, then

$$\begin{aligned} \rho _\mathbb {E}\left( \bigcap _{j=1}^n\left( U_{\eta _j} \cap \mathfrak {o}^d \right) \right) = \prod _{j=1}^n s_{\eta _j}, \end{aligned}$$

where again $$s_\eta = \nu _\eta (U_\eta ).$$

With the above corollary we are able to prove the following generalized version of Theorem 2.4, the main result of this paper.

### Theorem 3.5

Let *d* and *n* be positive integers. Let *K* be a number field with $k = [K: \mathbb {Q}]$, $\mathfrak {o}$ its ring of integers and $\mathbb {E}$ an integral basis of $\mathfrak {o}$. For each $\mathfrak {p} \in \mathcal {P}_K$, let $U_\mathfrak {p} \subseteq \mathfrak {o}_\mathfrak {p}^d$ be such that $\nu _\mathfrak {p}(\partial (U_\mathfrak {p})) =0$ and define $s_\mathfrak {p} = \nu _\mathfrak {p}(U_\mathfrak {p})$. Let $U_\infty \subseteq k_\infty ^d$, such that $\mu _\infty (\partial (U_\infty ))=0$ and $\mathbb {R}_{\ge 0} \cdot U_\infty = U_\infty$. We define $s_\infty = \frac{1}{2^{dk}}\nu _\infty \left( U_\infty \cap [-1,1] \cdot O(1, \mathbb {E})^d \right)$. If

4 $$\begin{aligned} \lim _{M \rightarrow \infty } \overline{\rho }_\mathbb {E} \left( \mathfrak {o}^d \cap \bigcup _{p>M} \bigcup _{\mathfrak {p}\in \mathcal {P}_K, \ \mathfrak {p} \vert p} U_\mathfrak {p} \right) = 0, \end{aligned}$$

is satisfied and for some $\alpha \in [0, \infty ]$ there exist absolute constants $c', c \in \mathbb {Z}$, such that for all $H\ge 1$ and for all $A \in O(H,\mathbb {E})_I^d$ one has that

5 $$\begin{aligned} \left| \left\{ \mathfrak {p}\in \mathcal {P}_K \mid \mathfrak {p} \succ c'H^\alpha , A \in U_\mathfrak {p} \cap O(H, \mathbb {E})_I^d \right\} \right| <c \end{aligned}$$

and that there exists a sequence $(v_\mathfrak {p})_{\mathfrak {p}\in \mathcal {P}_K}$, such that for all $m\in \{ 1, \dots , n\}$ and all $\mathfrak {p}_1, \dots , \mathfrak {p}_{{m}} \preceq c'H^\alpha$ with $\mathfrak {p_j}$ pairwise distinct one has that

6 $$\begin{aligned}{} & {} \left| \bigcap _{j=1}^{{m}} U_{\mathfrak {p}_j} \cap O(H, \mathbb {E})_I^d \right| \le (2H)^{kd} \prod \limits _{j=1}^{{m}} v_{\mathfrak {p}_j}, \end{aligned}$$

7 $$\begin{aligned}{} & {} \sum _{\mathfrak {p} \in \mathcal {P}_K} v_\mathfrak {p} \text {converges}, \end{aligned}$$

then it follows that

$$\begin{aligned} \mu _{n, \mathbb {E}}&= \lim \limits _{H \rightarrow \infty } \displaystyle {\frac{\sum \limits _{A \in O(H, \mathbb {E})_I^d } |\{ \eta \in N_K \mid A \in U_{\eta } \}|^n }{ (2H)^{kd}} } \end{aligned}$$

exists and $\mu _{n, \mathbb {E}}<\infty$. For $\tau \in \mathbb {N}^n$ with $\sum \limits _{j=1}^n j \tau _j=n$, we define $\ell (\tau ) = \sum \limits _{j=1}^n \tau _j$ and denote by $c(\tau )$ the number of partitions of $\{1, \dots , n\}$ which contain exactly $\tau _j$ sets of cardinality *j*. Then we have the formula

8 $$\begin{aligned} \mu _{n, \mathbb {E}} = \sum _{\tau \in \mathbb {N}^n, \ \sum _{j=1}^n j \tau _j =n} c(\tau ) \sum _{\begin{array}{c} \eta _1, \dots , \eta _{\ell (\tau )}\in N_K \\ \forall i<j \in \{1, \dots , \ell (\tau )\}, \ \eta _i \ne \eta _j \end{array}} \prod \limits _{m=1}^{\ell (\tau )} s_{\eta _m}, \end{aligned}$$

### Remark 3.6

Note that similarly to Remark 2.5 we get that the conditions are satisfied for all integral bases (with possibly different constants) as soon as they are satisfied for one particular integral basis. Also, the expression in (8) is independent of the integral basis for $U_\infty = \emptyset$ (as only $s_\infty$ depends on $\mathbb {E}$). Note that the dependence on $\mathbb {E}$ is not just a technicality, but reflects the fact that the basis alters the way the density of $U_\infty$ is measured (as it is a cone and not a lattice, its density is not invariant under $\mathbb {Z}$-module isomorphisms). We could easily develop our theorem in the same setting as in [3], where the box $O(H,\mathbb {E})^d$ is replaced by $H \Omega _\infty \cap \mathfrak {o}^d$ for some bounded set $\Omega _\infty \subset k_\infty ^d$ with $\mu _\infty (\partial \Omega _\infty )=0$ and $\mu _\infty (\Omega _\infty )>0$. The proof would be the same and the results would only differ by normalizing constants.

Finally, if the conditions of Theorem 3.5 are satisfied for some integer $n \ge 1$, then the same holds true for any positive integer $m\le n$.

### Proof

For $A\in \mathfrak {o}^d$ and $\eta \in N_K$, we define

$$\begin{aligned} \tau (A,\eta ) = {\left\{ \begin{array}{ll} 1,&{} A\in U_\eta ,\\ 0,&{} \text {else}. \end{array}\right. } \end{aligned}$$

For $M\in \mathbb {N},$ we have that

$$\begin{aligned} \sum \limits _{A\in O(H, \mathbb {E})_I^d} \frac{\left( \sum \limits _{\eta \in N_K} \tau (A,\eta ) \right) ^n}{(2H)^{kd}} =\sum \limits _{j=0}^n \left( {\begin{array}{c}n\\ j\end{array}}\right) R_j(M,H), \end{aligned}$$

where for all $j \in \{0, \ldots , n\}$, we define

$$\begin{aligned} R_j(M,H) = \sum _{A\in O(H, \mathbb {E})_I^d} \frac{\left( \sum \limits _{\mathfrak {p}\in \mathcal {P}_K, \ \mathfrak {p} \succ M} \tau (A,\mathfrak {p}) \right) ^{n-j} \left( \sum \limits _{\eta \in N_K, \ \eta \preceq M} \tau (A,\eta )\right) ^j}{(2H)^{kd}}. \end{aligned}$$

First we show that for all $j\in \{0, \dots , n-1\}$ the terms $R_j(M,H)$ are negligible for *M* going to infinity. We define

$$\begin{aligned} \ell _{A,H} = \left| \left\{ \mathfrak {p}\in \mathcal {P}_K \mid \mathfrak {p} \succ c'H^\alpha , A \in U_\mathfrak {p} \cap O(H, \mathbb {E})_I^d \right\} \right| . \end{aligned}$$

Then by (5) there exists $c>0$ such that for all $A\in \mathfrak {o}^d$ and all $H \ge 1$ holds $\ell _{A,H} \le c$. Thus, we get that

$$\begin{aligned} S_n&(M ,H):= \sum \limits _{A\in O(H, \mathbb {E})_I^d} \frac{\left( \sum \limits _{\mathfrak {p}\in \mathcal {P}_K, \ \mathfrak {p} \succ M} \tau (A,\mathfrak {p}) \right) ^n}{(2H)^{kd}} \\ =&\sum \limits _{i=0}^n \left( {\begin{array}{c}n\\ i\end{array}}\right) \sum \limits _{\begin{array}{c} A\in O(H, \mathbb {E})_I^d \\ A \in \bigcup _{\mathfrak {p}_1, \dots , \mathfrak {p}_n \succ M} \bigcap _{j=1}^n U_{\mathfrak {p}_j} \end{array}} \hspace{-1cm} \frac{ \vert \{ (\mathfrak {p}_j)_{j=1}^n \in \mathcal {P}_K^n \ \vert \ M \prec \mathfrak {p}_1, \dots , \mathfrak {p}_i \prec c'H^\alpha \prec \mathfrak {p}_{i+1}, \dots , \mathfrak {p}_n \} \vert }{(2H)^{kd}}. \end{aligned}$$

We split the sum into two parts and consider first the term with $i=0$, which can be estimated by

$$\begin{aligned} \ell _{A,H}^n \frac{\vert O(H, \mathbb {E})_I^d \cap \bigcup \limits _{\mathfrak {p}\in \mathcal {P}_K, \ \mathfrak {p} \succ M} U_\mathfrak {p} \vert }{(2H)^{kd}}. \end{aligned}$$

The remaining terms are bounded from above by

$$\begin{aligned} \sum _{i=1}^n \left( {\begin{array}{c}n\\ i\end{array}}\right)&\sum \limits _{\begin{array}{c} A\in O(H, \mathbb {E})_I^d \\ A \in \bigcup _{\mathfrak {p}_1, \dots , \mathfrak {p}_n \succ M} \bigcap _{j=1}^n U_{\mathfrak {p}_j} \end{array}} \hspace{-0.8 cm} \frac{\ell _{A,H}^{n-i} \vert \{ (\mathfrak {p}_j)_{j=1}^i \in \mathcal {P}_K^i \ \vert \ M \prec \mathfrak {p}_1, \dots , \mathfrak {p}_i \prec c'H^\alpha \} \vert }{(2H)^{kd}} \\ \le&\sum \limits _{i=1}^n \left( {\begin{array}{c}n\\ i\end{array}}\right) c^{n-i} \sum \limits _{\begin{array}{c} (\mathfrak {p}_1, \dots , \mathfrak {p}_i)\in \mathcal {P}_K^i \\ M \prec \mathfrak {p}_1, \dots , \mathfrak {p}_i \prec c'H^\alpha \end{array}} \hspace{-0.4cm} \frac{\vert O(H, \mathbb {E})_I^d \cap \bigcap \limits _{j=1}^i U_{\mathfrak {p}_j} \vert }{(2H)^{kd}}. \end{aligned}$$

We would like to use (6) to estimate the intersection. However, (6) only applies if the sets in the intersection are pairwise distinct. In order to account for this we need to count how many configurations of size *i* can give raise to configurations of size *j* with strictly increasing entries. For this we fix a set of $\{\mathfrak {q}_1, \dots , \mathfrak {q}_j\}\subseteq \mathcal {P}_K$ with $\mathfrak {q_1}\prec \mathfrak {q_2}\prec \dots \prec \mathfrak {q}_j$ and note that we have the following very crude estimate

$$\begin{aligned} \vert \{ (\mathfrak {p_1}, \dots , \mathfrak {p}_i)\in \mathcal {P}_K^i \ : \ \{ \mathfrak {p}_1, \dots , \mathfrak {p}_i\} = \{\mathfrak {q}_1, \dots , \mathfrak {q}_j\}\}\vert \le j^i, \end{aligned}$$

i.e., we just estimate by all possible combinations. Combining the last three inequalities with (6), we obtain

$$\begin{aligned} S_n(M,H) \le&c^{n} \frac{\vert O(H, \mathbb {E})_I^d \cap \bigcup \limits _{\mathfrak {p}\in \mathcal {P}_K, \ \mathfrak {p} \succ M} U_\mathfrak {p} \vert }{(2H)^{kd}} \\ {}&+ \sum \limits _{i=1}^n {\sum _{j=1}^i} \left( {\begin{array}{c}n\\ i\end{array}}\right) c^{n-i} j^i\sum \limits _{\begin{array}{c} (\mathfrak {q}_1, \dots , \mathfrak {q}_{ {j}})\in \mathcal {P}_K^{ {j}} \\ M \prec \mathfrak {q}_1 {\prec \dots \prec } \mathfrak {q}_{ {j}} \prec c'H^\alpha \end{array}} \left( \prod _{ {k=1}}^{ {j}} v_{\mathfrak {q}_{ {k}}} \right) \\ \le&c^{n} \frac{\vert O(H, \mathbb {E})_I^d \cap \bigcup \limits _{\mathfrak {p}\in \mathcal {P}_K, \ \mathfrak {p} \succ M} U_\mathfrak {p} \vert }{(2H)^{kd}} \\ {}&+ \sum \limits _{i=1}^n {\sum _{j=1}^i} \left( {\begin{array}{c}n\\ i\end{array}}\right) c^{n-i}j^i \left( \sum _{\mathfrak {p} \in \mathcal {P}_K, \ \mathfrak {p} \succ M} v_\mathfrak {p} \right) ^{ {j}}. \end{aligned}$$

This implies that

$$\begin{aligned} \limsup _{H\rightarrow \infty } S_n(M,H) \le&c^{n} \overline{\rho }_\mathbb {E}\left( \mathfrak {o}^d \cap \bigcup \limits _{\mathfrak {p}\in \mathcal {P}_K, \ \mathfrak {p} \succ M} U_\mathfrak {p} \right) \\ {}&+ \sum \limits _{i=1}^n {\sum _{j=1}^i } \left( {\begin{array}{c}n\\ i\end{array}}\right) c^{n-i} j^i \left( \sum _{\mathfrak {p} \in \mathcal {P}_K, \ \mathfrak {p} \succ M} v_\mathfrak {p} \right) ^{ {j}}. \end{aligned}$$

Thus, we get from (4) and (7)

9 $$\begin{aligned} \lim _{M \rightarrow \infty } \limsup _{H\rightarrow \infty } S_n(M,H) =0. \end{aligned}$$

On the other hand, (6) and (7) imply that

10 $$\begin{aligned} \begin{aligned} R_n(M,H)&=\sum _{A\in O(H, \mathbb {E})_I^d} \frac{\left( \sum \limits _{\eta \preceq M } \tau (A,\eta ) \right) ^n}{(2H)^{kd}} \\&= \sum \limits _{\eta _1, \dots , \eta _n \preceq M } \sum \limits _{A\in O(H, \mathbb {E})_I^d} \frac{\prod \limits _{j=1}^n \tau (A,\eta _j)}{(2H)^{kd}} \\&= \sum \limits _{\eta _1, \dots , \eta _n \preceq M } \frac{\vert O(H, \mathbb {E})_I^d \cap \bigcap \limits _{j=1}^n U_{\eta _j} \vert }{(2H)^{kd}} \\&\le {2^n \sum _{k=1}^n}\left( \sum \limits _{\eta \in N_K} v_\eta \right) ^{ {k}} <\infty . \end{aligned} \end{aligned}$$

For $j\in \{1, \dots , n-1\}$ we have by Hölder’s inequality with $p=\frac{n}{n-j}$ and $q=\frac{n}{j}$, that

$$\begin{aligned} R_j(M,H) \le S_n(M,H)^{(n-j)/n} R_n(M,H)^{j/n}. \end{aligned}$$

Thus, by (9) and (10) we get for all $j\in \{0, \dots , n-1\}$

$$\begin{aligned} \lim _{M \rightarrow \infty } \limsup _{H\rightarrow \infty } R_j(M,H) = 0. \end{aligned}$$

Hence, if we can show that $\lim \limits _{M \rightarrow \infty } \lim \limits _{H \rightarrow \infty } R_n(M,H)$ exists, then $\mu _{n, \mathbb {E}}$ exists as well. and we have

$$\begin{aligned} \mu _{n, \mathbb {E}} = \lim \limits _{M \rightarrow \infty } \lim \limits _{H\rightarrow \infty } R_n(M,H). \end{aligned}$$

For $\tau \in \mathbb {N}^n$ with $\sum \limits _{j=1}^n j \tau _j=n$, we define $\ell (\tau ) = \sum _{j=1}^n \tau _j$ and denote by $c(\tau )$ the number of partitions of $\{1, \dots , n\}$ which contain exactly $\tau _j$ sets of cardinality *j*. Using Corollary 3.4, we obtain

$$\begin{aligned}&\lim \limits _{M \rightarrow \infty } \lim \limits _{H \rightarrow \infty } R_n(M,H) \\&\quad = \lim \limits _{M \rightarrow \infty } \lim \limits _{H \rightarrow \infty } \sum \limits _{\eta _1, \dots , \eta _n \preceq M } \frac{\vert O(H, \mathbb {E})_I^d \cap \bigcap \limits _{j=1}^n U_{\eta _j} \vert }{(2H)^{kd}} \\&\quad = \lim \limits _{M \rightarrow \infty } \lim \limits _{H \rightarrow \infty } \sum \limits _{\tau \in \mathbb {N}^n, \ \sum _{j=1}^n j \tau _j =n} c(\tau ) \sum \limits _{\begin{array}{c} \eta _1, \dots , \eta _{\ell (\tau )} \preceq M \\ \forall i<j\in \{1, \dots , \ell (\tau )\}, \ \eta _i \ne \eta _j \end{array}} \frac{\vert O(H, \mathbb {E})_I^d \cap \bigcap \limits _{j=1}^{\ell (\tau )} U_{\eta _j} \vert }{(2H)^{kd}} \\&\quad = \lim \limits _{M \rightarrow \infty } \sum \limits _{\tau \in \mathbb {N}^n, \ \sum \limits _{j=1}^n j \tau _j =n} c(\tau ) \sum \limits _{\begin{array}{c} \eta _1, \dots , \eta _{\ell (\tau )}\preceq M \\ \forall i<j\in \{1, \dots , \ell (\tau )\}, \ \eta _i \ne \eta _j \end{array}} \rho _\mathbb {E} \left( \bigcap \limits _{j=1}^{\ell (\tau )} U_{\eta _j} \cap \mathfrak {o}^d \right) \\&\quad = \lim \limits _{M \rightarrow \infty } \sum \limits _{\tau \in \mathbb {N}^n, \ \sum \limits _{j=1}^n j \tau _j =n} c(\tau ) \sum \limits _{\begin{array}{c} \eta _1, \dots , \eta _{\ell (\tau )} \preceq M \\ \forall i<j\in \{1, \dots , \ell (\tau )\}, \ \eta _i \ne \eta _j \end{array}} \prod \limits _{j=1}^{\ell (\tau )} s_{\eta _j} \\&\quad =\sum \limits _{\tau \in \mathbb {N}^n,\ \sum _{j=1}^n j \tau _j =n} c(\tau ) \sum \limits _{\begin{array}{c} \eta _1, \dots , \eta _{\ell (\tau )} \in N_K \\ \forall i<j\in \{1, \dots , \ell (\tau )\} , \ \eta _i \ne \eta _j \end{array}} \prod \limits _{j=1}^{\ell (\tau )} s_{\eta _j}. \end{aligned}$$

This proves (8). Note that this last expression is finite. We have the crude estimate

$$\begin{aligned} \mu _{n, \mathbb {E}} \le 2^n n^n \left( 1+ \sum \limits _{\eta \in N_K} s_\eta \right) ^n < \infty \end{aligned}$$

by (7). $$\square $$

### Remark 3.7

The conditions of Theorem 2.4 also allow to conclude the existence of all central moments up to order *n*. For $j\in \{1, \dots , n\}$ we have

$$\begin{aligned} \lim \limits _{H \rightarrow \infty } \displaystyle {\frac{\sum \limits _{A \in O(H, \mathbb {E})_I^d } \left( |\{ \eta \in N_K \mid A \in U_{\eta } \} |- \mu _\mathbb {E}\right) ^n }{ (2H)^{kd}} } = \sum _{j=0}^n \left( {\begin{array}{c}n\\ j\end{array}}\right) (-\mu _{\mathbb {E}})^{n-j} \mu _{j, \mathbb {E}}. \end{aligned}$$

Now we briefly compute the variance

$$\begin{aligned} \sigma ^2_\mathbb {E} = \lim \limits _{H \rightarrow \infty } \displaystyle {\frac{\sum \limits _{A \in O(H, \mathbb {E})_I^d } (|\{ \eta \in N_K \mid A \in U_{\eta } \} |- \mu _\mathbb {E})^2 }{ (2H)^{kd}} }. \end{aligned}$$

For $n=2$ we have two $\tau \in \mathbb {N}^2$ such that $\tau _1+\tau _2 =2$, namely $\tau ^{(1)}= (2,0)$ and $\tau ^{(2)} = (0,1)$. One readily computes $c\left( \tau ^{(1)}\right) =1, \ell \left( \tau ^{(1)}\right) =2$ and $c\left( \tau ^{(2)}\right) =1, \ell \left( \tau ^{(2)}\right) =1$. Thus, we get

$$\begin{aligned} \mu _{2, \mathbb {E}} = \sum _{\eta _1, \eta _2 \in N_K,\ \eta _1 \ne \eta _2 } s_{\eta _1} s_{\eta _2} + \sum _{\eta \in N_K} s_\eta = \mu _\mathbb {E}^2 - \sum _{\eta \in N_K} s_\eta ^2 + \mu _\mathbb {E}. \end{aligned}$$

Hence, we obtain

$$\begin{aligned} \begin{aligned} \sigma ^2_\mathbb {E} = \mu _{2, \mathbb {E}} - 2 \mu _\mathbb {E}^2 + \mu _\mathbb {E}^2 = \mu _\mathbb {E} - \sum _{\eta \in N_K} s_\eta ^2. \end{aligned} \end{aligned}$$

Also for the variance we can restrict to subsets $T \subseteq \mathfrak {o}^d,$ as

$$\begin{aligned} \sigma ^2_{T, \mathbb {E}} = \lim \limits _{H \rightarrow \infty } \displaystyle {\frac{\sum \limits _{A \in O(H, \mathbb {E})_I^d \cap T } (\mid \{ \eta \in N_K \mid A \in U_{\eta } \} \mid - \mu _\mathbb {E})^2 }{ \mid O(H, \mathbb {E})^d \cap T \mid } }. \end{aligned}$$

Due to Lemma 3.3, if $T^C \subseteq P^{-1}(\{ \emptyset \})$ and the density of *T* is non-zero, we get

$$\begin{aligned} \sigma ^2_T = \frac{1}{\rho (T)}\left( \mu ^2 - \sum \limits _{\eta \in N_K} s_\eta ^2 + \mu \right) - \frac{2\mu _T}{\rho (T)} \mu + \mu _T^2. \end{aligned}$$

## Applications

### Density computations

In this section, we compute the densities of Eisenstein and shifted Eisenstein polynomials over number fields, using Theorem 2.4.

#### Definition 4.1

Let $\mathfrak {p}$ be a non-zero prime ideal of $\mathfrak {o}$. A polynomial $f(x) \in \mathfrak {o}[x]$ of degree *d* represented by the tuple $(a_0, \ldots , a_{d-1}, a_d) \in \mathfrak {o}^{d+1}$ is said to be $\mathfrak {p}$-Eisenstein if

$$\begin{aligned} a_d \not \in \mathfrak {p}, \ a_0 \not \in \mathfrak {p}^2 \text { and} \ a_i \in \mathfrak {p}\; \forall i \in \{0, \ldots , d-1\} . \end{aligned}$$

In addition, *f* is said to be Eisenstein if there exists a prime ideal $\mathfrak {p}$ of $\mathfrak {o}$ such that *f*(*x*) is $\mathfrak {p}$-Eisenstein.

Denote by ${\Gamma _\mathfrak {p}}^d$ the set of all $\mathfrak {p}$-Eisenstein polynomials of degree *d* and by $\Gamma ^d$ the set of all Eisenstein polynomials of degree *d*. We choose

11 $$\begin{aligned} U_\mathfrak {p}= (\mathfrak {p}\mathfrak {o}_\mathfrak {p}\setminus \mathfrak {p}^2 \mathfrak {o}_\mathfrak {p}) \times (\mathfrak {p}\mathfrak {o}_\mathfrak {p})^{d-1} \times (\mathfrak {o}_\mathfrak {p}\setminus \mathfrak {p}\mathfrak {o}_\mathfrak {p}) \subseteq \mathfrak {o}_\mathfrak {p}^{d+1} \end{aligned}$$

and $U_\infty =\emptyset$. Note that we have

12 $$\begin{aligned} {\Gamma _\mathfrak {p}}^d = (\mathfrak {p}\setminus \mathfrak {p}^2) \times \mathfrak {p}^{d-1} \times (\mathfrak {o}\setminus \mathfrak {p}) = \mathfrak {o}^{d+1} \cap U_\mathfrak {p}. \end{aligned}$$

By abuse of notation, we will use the same symbol for an element of $\mathfrak {o}$ and its image in the ring of integers $\mathfrak {o}_\mathfrak {p}$ in the completion $K_\mathfrak p$.

#### Corollary 4.2

Let *K* be a number field, $\mathfrak {o}$ its ring of integers and let $d\ge 2$ be an integer. The density of the set $\Gamma ^d$ of Eisenstein polynomials of degree *d* over $\mathfrak {o}$ is given by

13 $$\begin{aligned} \rho \left( \Gamma ^d\right) = 1-\prod \limits _{\mathfrak {p}\in \mathcal {P}_K}\left( 1-\frac{(N(\mathfrak {p})-1)^2}{N(\mathfrak {p})^{d+2}}\right) , \end{aligned}$$

where $$N(\mathfrak {p})= \vert \mathfrak {o}/\mathfrak {p} \vert = p^{\deg (\mathfrak {p})}$$, and $$\deg (\mathfrak {p}) = [ \mathfrak {o}/ \mathfrak {p}: \mathbb {F}_p]$$.

#### Proof

Recall the expressions for the sets ${\Gamma _\mathfrak {p}}^d$ and $U_\mathfrak {p}$ from equations (12) and (11). With the system of sets $(U_\eta )_{\eta \in N_K}$, consider the map *P* defined as in Theorem 2.4. Note that we have

$$\begin{aligned}P^{-1}(\{\emptyset \}) = \{a \in \mathfrak {o}^{d+1} \mid a\not \in U_\mathfrak {p}\ \forall \mathfrak {p}\in \mathcal {P}_K\},\end{aligned}$$

thus

$$\begin{aligned}P^{-1}(\{\emptyset \})^C = \{a \in \mathfrak {o}^{d+1} \mid \ a \in \Gamma _{\mathfrak {p}}^{d} \ \text { for some } \mathfrak {p}\in \mathcal {P}_K\} = \Gamma ^d.\end{aligned}$$

In other words, the system $(U_\eta )_{\eta \in N_K}$ corresponds (as per Definition 3.2) to the set $\Gamma ^d$ of Eisenstein polynomials of degree *d*. We clearly have that $\partial (U_\mathfrak {p}) =\emptyset$. Hence, in order to apply Theorem 2.4 to this system, we only have to check (2).

Letting $f(x_1, \dots , x_{d+1})=x_1$ and $g(x_1, \dots , x_{d+1})=x_2$, we see that

$$\begin{aligned} S_M(f,g) = \bigcup \limits _{\mathfrak {p}\succ M} \left( \mathfrak {p}\times \mathfrak {p}\times \mathfrak {o}^{d-1} \right) , \end{aligned}$$

and

$$\begin{aligned} \mathfrak {o}^{d+1} \bigcap \left( \bigcup \limits _{\mathfrak {p}\succ M} U_\mathfrak {p}\right) = \bigcup \limits _{\mathfrak {p}\succ M} \Gamma _\mathfrak {p}^d \subseteq S_M(f,g). \end{aligned}$$

Thus, the application of Lemma 2.6 gives Condition (2) in this case. One easily computes that

$$\begin{aligned} \nu _\mathfrak {p}(U_\mathfrak {p})= \dfrac{(p^{\deg (\mathfrak {p})}-1)^2}{p^{\deg (\mathfrak {p})(d+2)}}. \end{aligned}$$

Hence, applying Theorem 2.4 yields

$$\begin{aligned} \rho (\Gamma ^d)&= 1-m(\{\emptyset \}) = 1-\prod \limits _{\eta \in \emptyset } s_\eta \prod \limits _{\eta \not \in \emptyset }(1-s_\eta ) \\&=1- \prod \limits _{\mathfrak {p}\in \mathcal {P}_K}(1-\nu _{\mathfrak {p}}(U_\mathfrak {p})) \\&= 1-\prod \limits _{\mathfrak {p}\in \mathcal {P}_K}\left( 1-\dfrac{(p^{\deg (\mathfrak {p})}-1)^2}{p^{\deg (\mathfrak {p})(d+2)}}\right) \\&=1-\prod \limits _{\mathfrak {p}\in \mathcal {P}_K}\left( 1-\dfrac{(N(\mathfrak {p})-1)^2}{N(\mathfrak {p})^{d+2}}\right) . \end{aligned}$$

$$\square $$

#### Definition 4.3

Let $\mathfrak {p}$ be a non-zero prime ideal in $\mathfrak {o}$ and $f \in \mathfrak {o}[x]$ be a monic polynomial of degree *d*. We call *f* a shifted $\mathfrak {p}$-Eisenstein polynomial if there exists some $b \in \mathfrak {o}$, such that $f(x+b)$ is $\mathfrak {p}$-Eisenstein. In addition, *f* is said to be a shifted Eisenstein polynomial if there exists a prime ideal $\mathfrak {p}$ such that *f* is a shifted $\mathfrak {p}$-Eisenstein polynomial.

We will denote by $\widetilde{\Gamma }^d$ and $\widetilde{\Gamma }_\mathfrak {p}^d$ the set of all shifted Eisenstein polynomials, respectively shifted $\mathfrak {p}$-Eisenstein polynomials, of degree *d*. We will identify elements of $\mathfrak {o}_\mathfrak {p}^{d+1}$ with monic polynomials of degree *d* over $\mathfrak {o}_\mathfrak {p}$.

For $b \in \mathfrak {o}_\mathfrak {p}^{d+1}$, we will denote by $\sigma _b$ the following map:

$$\begin{aligned}\sigma _b : \mathfrak {o}_\mathfrak {p}^{d+1}&\rightarrow \mathfrak {o}_\mathfrak {p}^{d+1}, \\ f(x)&\mapsto f(x+b). \end{aligned}$$

Note that $\sigma _b$ is an automorphism with inverse $\sigma _{-b}$. It is also clearly continuous and linear. Moreover, for any $b\in \mathfrak {o}$ we have that $\mathfrak {o}^{d+1}$ is invariant under $\sigma _b$, in fact $\sigma _b(\mathfrak {o}^{d+1}) = \mathfrak {o}^{d+1}$. By abuse of notation, for $b \in \mathfrak {o}$ we also denote the restricted maps $\mathfrak {o}^{d+1} \rightarrow \mathfrak {o}^{d+1}$ by $\sigma _b$.

The following two results were developed following the methods in [17].

#### Proposition 4.4

Let $f(x) \in \mathfrak {o}[x]$ be a polynomial of degree *d* and *b* denote any element of $\mathfrak {o}$. Suppose that *f*(*x*) is $\mathfrak {p}$-Eisenstein. Then $f(x+b)$ is $\mathfrak {p}$-Eisenstein if and only if $b \in \mathfrak {p}$.

#### Proof

We first write

$$\begin{aligned} f(x) = a_0 +a_1x + \cdots + a_{d-1}x^{d-1} +a_d x^d. \end{aligned}$$

For $b \in \mathfrak {p}$, we have that

$$\begin{aligned} f (x+b)&=a_0+ a_1(x+b) + \cdots + a_{d-1}(x+b)^{d-1} + a_d (x+b)^d = \sum _{i=0}^d a_i' x^i, \end{aligned}$$

where $$a_i' = \sum _{j=i}^d \left( {\begin{array}{c}j\\ i\end{array}}\right) a_j b^{j-i}.$$ Since *f* is $\mathfrak {p}$-Eisenstein, we must have that $a_j \equiv 0 \mod \mathfrak {p}$ for all $j \in \{0, \ldots , d-1\}$, $a_d \not \equiv 0 \mod \mathfrak {p}$, and $a_0 \not \equiv 0 \mod \mathfrak {p}^2$. It follows directly that $$a_i^\prime \equiv \left( {\begin{array}{c}d\\ i\end{array}}\right) a_d b^{d-i} \mod \mathfrak {p}$$ for all $i \in \{0, \ldots , d-1\}$ and $a_d'=a_d$. Thus, $f(x+b)$ is $\mathfrak {p}$-Eisenstein if and only if

$$\begin{aligned} \left( {\begin{array}{c}d\\ i\end{array}}\right) b^{d-i}\equiv 0 \mod \mathfrak {p}\; \forall \; 0\le i < d, \; \text {and} \; a_0' = \sum _{j=0}^d a_j b^j \not \equiv 0 \mod \mathfrak {p}^2, \end{aligned}$$

which is equivalent to

$$\begin{aligned} b \equiv 0 \mod \mathfrak {p}, \; \text {and} \; {a_0}^\prime = \sum _{j=0}^d a_j b^j \not \equiv 0 \mod \mathfrak {p}^2. \end{aligned}$$

Note, that if $b \equiv 0 \mod \mathfrak {p}$, we get ${a_0}^\prime \equiv a_0 \not \equiv 0 \mod \mathfrak {p}^2$ as $a_1\in \mathfrak {p}$ since *f* is $\mathfrak {p}$-Eisenstein. We have thus shown that $f(x+b)$ is $\mathfrak {p}$-Eisenstein if and only if $b \equiv 0 \mod \mathfrak {p}$, as required. $\square$

#### Corollary 4.5

Let $b_1$ and $b_2$ be elements of $\mathfrak {o}$ such that $b_1 \not \equiv b_2 \mod \mathfrak {p}$. Then

$$\begin{aligned} \sigma _{b_1}^{-1}(\Gamma _\mathfrak {p}^d) \cap \sigma _{b_2}^{-1}(\Gamma _\mathfrak {p}^d) = \emptyset . \end{aligned}$$

#### Proof

Let $b=b_2-b_1$, then it is easy to see that the statement of the corollary is equivalent to

$$\begin{aligned} \ \Gamma _\mathfrak {p}^d \cap \sigma _b^{-1}(\Gamma _\mathfrak {p}^d) =\emptyset . \end{aligned}$$

The claim now follows immediately from Proposition 4.4. $\square$

#### Theorem 4.6

Let $\mathfrak {p}$ be a non-zero prime ideal of $\mathfrak {o}$. We have the following decomposition for the set $\widetilde{\Gamma }_\mathfrak {p}^d$ of all shifted $\mathfrak {p}$-Eisenstein polynomials of degree *d*:

$$\begin{aligned}\widetilde{\Gamma }_\mathfrak {p}^d = \bigsqcup _{b+\mathfrak {p}\in \mathfrak {o}/ \mathfrak {p}} \sigma _b^{-1}\left( \Gamma _\mathfrak {p}^d\right) , \end{aligned}$$

where $b+\mathfrak {p}$ is an element in $\mathfrak {o}/\mathfrak {p}$.

#### Proof

The set of all shifted $\mathfrak {p}$-Eisenstein polynomials of degree *d* can be written as the union

14 $$\begin{aligned} \bigcup _{b\in \mathfrak {o}} \sigma _b^{-1}(\Gamma _\mathfrak {p}^d) = \bigsqcup _{b+\mathfrak {p}\in \mathfrak {o}/\mathfrak {p}} \bigcup _{c \in b+\mathfrak {p}} \sigma _c^{-1}(\Gamma _\mathfrak {p}^d), \end{aligned}$$

where the fact that the first union is disjoint follows from Corollary 4.5. We also have, from Proposition 4.4, that for all $c_1$ and $c_2$ satisfying $c_1 \equiv c_2 \mod \mathfrak {p}$,

$$\begin{aligned}\sigma _{c_1}^{-1}(\Gamma _\mathfrak {p}^d) \cap \sigma _{c_2}^{-1}(\Gamma _\mathfrak {p}^d) = \sigma _{c_1}^{-1}(\Gamma _\mathfrak {p}^d) = \sigma _{c_2}^{-1}(\Gamma _\mathfrak {p}^d). \end{aligned}$$

Thus, the latter union in (14) is equal to any one of the sets, and we may write

$$\begin{aligned}\widetilde{\Gamma }_\mathfrak {p}^d = \bigsqcup _{b+\mathfrak {p}\in \mathfrak {o}/ \mathfrak {p}} \sigma _b^{-1}(\Gamma _\mathfrak {p}^d), \end{aligned}$$

where $b+\mathfrak {p}$ is an element in $\mathfrak {o}/\mathfrak {p}$. $\square$

#### Corollary 4.7

Let *K* be a number field, $\mathfrak {o}$ its ring of integers and let $d\ge 2$ be an integer. The density of the shifted Eisenstein polynomials of degree *d* over $\mathfrak {o}$ is given by

$$\begin{aligned} \rho \left( \widetilde{\Gamma }^d\right) = 1-\prod \limits _{\mathfrak {p}\in \mathcal {P}_K}\left( 1- \frac{(N(\mathfrak {p})-1)^2}{N(\mathfrak {p})^{d+1}}\right) ,\end{aligned}$$

where $$N(\mathfrak {p})= \vert \mathfrak {o}/\mathfrak {p} \vert = p^{\deg (\mathfrak {p})}$$ and $$\deg (\mathfrak {p}) = [ \mathfrak {o}/ \mathfrak {p}: \mathbb {F}_p]$$.

#### Proof

We have,

$$\begin{aligned}{} & {} \widetilde{\Gamma }_{\mathfrak {p}}^{d} = \bigsqcup _{b+\mathfrak {p}\in \mathfrak {o}/ \mathfrak {p}} \sigma _b^{-1}\left( (\mathfrak {p} \setminus \mathfrak {p}^2 )\times \mathfrak {p}^{d-1} \times \mathfrak {o}\setminus \mathfrak {p}\right) \\{} & {} \quad = \bigsqcup _{b+\mathfrak {p}\in \mathfrak {o}/ \mathfrak {p}} \sigma _b^{-1}\left( {\Gamma _\mathfrak {p}}^d\right) , \end{aligned}$$

where $b+\mathfrak {p}$ is an element in $\mathfrak {o}/\mathfrak {p}$. Let $U_\mathfrak {p}$ be defined as before.

We set

$$\begin{aligned} \widetilde{U}_\mathfrak {p}= \bigsqcup \limits _{b+\mathfrak {p}\in \mathfrak {o}/ \mathfrak {p}} \sigma _b^{-1} \left( U_\mathfrak {p}\right) \subset {\mathfrak {o}_\mathfrak {p}}^{d+1}, \end{aligned}$$

and $$\widetilde{U}_\infty = \emptyset $$.

As noted before, $\sigma _b(\mathfrak {o}^{d+1})= \mathfrak {o}^{d+1}$ for any $b\in \mathfrak {o}$ and thus, we have $\widetilde{\Gamma }_\mathfrak {p}^d = \mathfrak {o}^{d+1} \cap \widetilde{U}_\mathfrak {p}$. Now, with the system of sets $\widetilde{U}_\mathfrak {p}$ and the map *P* defined as in Theorem 2.4, we have, as before, that $P^{-1}(\{\emptyset \})^C$ is the set $\widetilde{\Gamma }^d$ of shifted Eisenstein polynomials of degree *d*. Since $\mathfrak {o}_\mathfrak {p}$ is compact, and ${\sigma _b}$ is a surjective and continuous endomorphism of $\mathfrak {o}_\mathfrak {p}^{d+1}$, we have that for any $b \in \mathfrak {o}$, $\sigma _b$ preserves the Haar measure. Thus,

$$\begin{aligned}{} & {} \nu _{\mathfrak {p}}(\widetilde{U}_\mathfrak {p}) = \nu _\mathfrak {p}\left( \bigsqcup _{b+\mathfrak {p}\in \mathfrak {o}/ \mathfrak {p}} \sigma _b^{-1}\left( U_\mathfrak {p}\right) \right) \\{} & {} \quad = \left|{\mathfrak {o}}/{\mathfrak {p}}\right|\nu _{\mathfrak {p}}({U}_\mathfrak {p})= p^{\deg (\mathfrak {p})}\cdot \nu _{\mathfrak {p}}({U}_\mathfrak {p}). \end{aligned}$$

We first deal with the case $d\ge 3$ and verify condition (2) for the system $(\widetilde{U}_\eta )_{\eta \in N_K}$. For this, we define polynomials $F, G \in \mathfrak {o}[x_0, \ldots , x_{d}]$ as

$$\begin{aligned} F(x_0, \ldots , x_{d})&= d^2 x_0 x_{d} - x_1 x_{d-1},\\ G(x_0, \ldots , x_{d})&= x_2 x_{d-2} - \left( {\begin{array}{c}d\\ 2\end{array}}\right) ^2 x_0 x_{d}. \end{aligned}$$

Clearly, these are coprime for any value of $d\ge 3$. We claim that

15 $$\begin{aligned}{} & {} \mathfrak {o}^{d+1} \bigcap \left( \bigcup \limits _{\mathfrak {p}\succ M} \widetilde{U}_\mathfrak {p}\right) \nonumber \\{} & {} \quad = \bigcup \limits _{\mathfrak {p}\succ M} \widetilde{\Gamma }_\mathfrak {p}^d \subseteq S_M(F,G). \end{aligned}$$

Let $a^\prime =(a_0^\prime , a_1^\prime , \ldots , a_{d}^\prime ) \in \widetilde{\Gamma }_\mathfrak {p}^d$ for some $\mathfrak {p}\succ M$. Then, there exists $b \in \mathfrak {o}$ such that $\sigma _{-b}(a^\prime )=a=(a_0, \dots , a_d)$ is $\mathfrak {p}$-Eisenstein. This is equivalent to saying $a'=\sigma _b(a)$ and hence we have the relations $$a_j^\prime = \sum _{i=j}^d \left( {\begin{array}{c}i\\ j\end{array}}\right) a_i b^{i-j}$$, $a_i \equiv 0 \mod \mathfrak {p}$ for $i\in \{0, \dots , d-1\}$ and $a_d \not \in \mathfrak {p}$. Thus, $$a_j^\prime = \left( {\begin{array}{c}d\\ j\end{array}}\right) a_d b^{d-j} \mod \mathfrak {p}$$. Clearly, $$a_j^\prime a_{d-j}^\prime \equiv \left( {\begin{array}{c}d\\ j\end{array}}\right) ^2 a_d^2 b^d \mod \mathfrak {p}$$, and in particular

$$\begin{aligned} a_0^\prime a_{d}^\prime&\equiv a_d^2 b^d \mod \mathfrak {p}, \\ a_1^\prime a_{d-1}^\prime&\equiv a_d^2 b^d d^2 \mod \mathfrak {p}, \\ a_2^\prime a_{d-2}^\prime&\equiv a_d^2 b^d \left( {\begin{array}{c}d\\ 2\end{array}}\right) ^2 \mod \mathfrak {p}. \end{aligned}$$

Therefore, we get

$$\begin{aligned} F(a^\prime ) \equiv G(a^\prime ) \equiv 0 \mod \mathfrak {p}\end{aligned}$$

or in other words $a^\prime \in S_M(F,G)$. This proves (15).

Finally, from Lemma 2.6 we have

$$\begin{aligned} \lim _{M \rightarrow \infty } \bar{\rho }_\mathbb {E}(S_M(F,G)) = 0 \end{aligned}$$

and thus

$$\begin{aligned} \lim _{M \rightarrow \infty } \bar{\rho }_\mathbb {E}\left( \mathfrak {o}^{d+1} \bigcap \left( \bigcup \limits _{\mathfrak {p}\succ M} \widetilde{U}_\mathfrak {p}\right) \right) = 0. \end{aligned}$$

Thus, applying Theorem 2.4 and using (11), we get

$$\begin{aligned} \rho \left( \widetilde{\Gamma }^d\right) = 1-m(\{\emptyset \})&= 1-\prod \limits _{\mathfrak {p}\in \mathcal {P}_K}(1-\nu _{\mathfrak {p}}(\widetilde{U}_\mathfrak {p})) \\&= 1-\prod \limits _{\mathfrak {p}\in \mathcal {P}_K}\left( 1-p^{\deg (\mathfrak {p})}\cdot \dfrac{(p^{\deg (\mathfrak {p})}-1)^2}{p^{\deg (\mathfrak {p})(d+2)}}\right) \\&=1-\prod \limits _{\mathfrak {p}\in \mathcal {P}_K}\left( 1- \dfrac{(N(\mathfrak {p})-1)^2}{N(\mathfrak {p})^{d+1}}\right) . \end{aligned}$$

Now we turn to the case $d=2$. We use the same strategy as in [17, Prop. 10] and fix some positive integer *M* and some integral basis $\mathbb {E}$ of $\mathfrak {o}$. Then we consider the system

$$\begin{aligned} V_\mathfrak {p} = {\left\{ \begin{array}{ll} \widetilde{U}_\mathfrak {p},&{} \mathfrak {p}\preceq M,\\ \emptyset ,&{} \text {else}, \end{array}\right. } \end{aligned}$$

and $V_\infty = \emptyset$. The system $(V_\eta )_{\eta \in N_K}$ clearly satisfies the conditions of Theorem 2.4 and thus

16 $$\begin{aligned}&1\ge \underline{\rho }_\mathbb {E}(\widetilde{\Gamma }^2) \nonumber \\&\quad \ge \underline{\rho }_\mathbb {E}\left( \bigcup _{\eta \in N_K} V_\eta \cap \mathfrak {o}^{3} \right) \nonumber \\&\quad = 1 - \prod _{\mathfrak {p}\in \mathcal {P}_K, \ \mathfrak {p}\preceq M}\nonumber \\&\quad \left( 1 - \frac{(N(\mathfrak {p})-1)^2}{N(\mathfrak {p})^3} \right) \nonumber \\&\quad \ge 1 - \prod _{\mathfrak {p}\in \mathcal {P}_K, \ \mathfrak {p}\preceq M} \left( 1 - \frac{1}{4N(\mathfrak {p})} \right) . \end{aligned}$$

By [13, Ch. VIII, Theorem 6] the series $\sum _{\mathfrak {p}\in \mathcal {P}_K} N(\mathfrak {p})^{-1}$ diverges to infinity. Hence, the product of (16) goes to zero for $M \rightarrow \infty$. Thus, we get

$$\begin{aligned}{} & {} \rho (\widetilde{\Gamma }^2)=1 = 1- \prod _{\mathfrak {p}\in \mathcal {P}_K} \nonumber \\{} & {} \quad \left( 1- \frac{(N(\mathfrak {p})-1)^2}{N(\mathfrak {p})^{3}} \right) . \end{aligned}$$

$$\square $$

### Computations of Higher Moments

In this section, we will apply Theorem 3.5 to compute the expected value and the variance of non-zero prime ideals $\mathfrak {p} \subset \mathfrak {o}$ over a general number field *K* such that a polynomial of degree *d* is $\mathfrak {p}$-Eisenstein. A minor modification yields the same claim for shifted Eisenstein polynomials.

#### Corollary 4.8

Let $d\ge 2$ be an integer and let *K* be number field and $\Gamma ^d$ be the Eisenstein polynomials of degree *d*. We associate the system $U_\mathfrak {p}= (\mathfrak {p}\mathfrak {o}_\mathfrak {p}{\setminus } \mathfrak {p}^2 \mathfrak {o}_\mathfrak {p}) \times (\mathfrak {p} \mathfrak {o}_\mathfrak {p})^{d-1} \times (\mathfrak {o}_\mathfrak {p}{\setminus } \mathfrak {p} \mathfrak {o}_\mathfrak {p})$ and $U_\infty = \emptyset$. This system satisfies the conditions of Theorem 3.5 for any $n\in \mathbb {N}$.

In particular, we have

$$\begin{aligned} \rho \left( \Gamma ^d\right)&= \left( 1-\prod \limits _{\mathfrak {p}\in \mathcal {P}_K}\left( 1-\frac{(N(\mathfrak {p})-1)^2}{N(\mathfrak {p})^{d+2}}\right) \right) , \\ \mu&= \sum \limits _{\mathfrak {p}\in \mathcal {P}_K}\frac{(N(\mathfrak {p})-1)^2}{N(\mathfrak {p})^{d+2}}, \quad \mu _{\Gamma ^d} = \rho (\Gamma ^d)^{-1} \mu , \end{aligned}$$

where $$N(\mathfrak {p})= \vert \mathfrak {o}/\mathfrak {p} \vert = p^{\deg (\mathfrak {p})}$$ and $$\deg (\mathfrak {p}) = [ \mathfrak {o}/ \mathfrak {p}: \mathbb {F}_p]$$.

Furthermore, the restricted variance is given by

$$\begin{aligned} \sigma ^2_{\Gamma ^d} = \frac{1}{\rho (\Gamma ^d)}\left( \mu ^2 - \sum \limits _{\eta \in \mathcal {P}_K} \left( \frac{(N(\mathfrak {p})-1)^2}{N(\mathfrak {p})^{d+2}}\right) ^2 + \mu \right) - \frac{2\mu _{\Gamma ^d}}{\rho (\Gamma ^d)} \mu + \mu _{\Gamma ^d}^2. \end{aligned}$$

#### Proof

We start by noting that $\rho (\Gamma ^d)$ was already computed in (13).

As $\mathfrak {o}$ is a Dedekind domain, we get that the intersection of infinitely many prime ideals in $\mathfrak {o}$ is the zero ideal. In particular, we have that the intersection of infinitely many ideals of the form $\mathfrak {p} \setminus \mathfrak {p}^2$ must be the empty set and hence, $I=\emptyset$ and $O(H,\mathbb {E})_I = O(H, \mathbb {E})$.

We need to check the assumptions of Theorem 3.5. Condition (4) follows directly from Lemma 2.6 applied to the polynomials $f(x_0, \dots , x_d)=x_0$ and $g(x_0, \dots , x_d)=x_1$.

Next we show that (5) is satisfied for any $\alpha >0$. Let $A=(A_0, \dots , A_d)\in \mathfrak {o}^{d+1}$, then we define $f_A(x)= \sum _{j=0}^d A_{d-j} x^j$ and we denote by ${\text {disc}}(f_A)$ the discriminant of $f_A$. If $\{ \mathfrak {p} \in \mathcal {P}_K \ \mid \ A \in U_\mathfrak {p} \} = \emptyset$, then (5) is trivially satisfied. Thus, we will now assume that $\{ \mathfrak {p} \in \mathcal {P}_K \ \mid \ A \in U_\mathfrak {p} \} \ne \emptyset$.

As the discriminant is the Sylvester matrix of the resultant of $f_A$ and $f_A'$, we get (as $A_d$ will always be multiplied by some $A_j$ with $j\in \{0, \dots , d-1\}$)

$$\begin{aligned} {\text {disc}}(f_A) \in \bigcap _{\mathfrak {p}\in \mathcal {P}_K, A \in U_\mathfrak {p}} \mathfrak {p}. \end{aligned}$$

Combining this with the observation that ${\text {disc}}(f_A)\ne 0$ as $f_A$ is irreducible (as *A* is contained in some $U_\mathfrak {p}$, $f_A$ satisfies the criterion of Eisenstein), we get

17 $$\begin{aligned} \begin{aligned} \vert N_{K/\mathbb {Q}}({\text {disc}}(f_A)) \vert&= N(\langle {\text {disc}}(f_A)\rangle _{\mathfrak {o}}) \\&\ge \prod _{\mathfrak {p}\in \mathcal {P}_K, \ A \in U_\mathfrak {p}} N(\mathfrak {p}) \ge H^{\alpha \vert \{ \mathfrak {p}\in \mathcal {P}_K \ \mid \ \mathfrak {p}\succeq H^\alpha , A \in U_\mathfrak {p} \} \vert }, \end{aligned} \end{aligned}$$

where we denote by *N* the absolute norm and $N_{K/\mathbb {Q}}$ the ideal norm on $\mathfrak {o}$.

On the other hand, using that the resultant is a homogeneous polynomial of degree $2d-2$ and $N_{K/\mathbb {Q}}({\text {disc}}(f_A)) = {\text {det}}_\mathbb {Z}(\mathfrak {o}\rightarrow \mathfrak {o}, x \mapsto {\text {disc}}(f_A)x)$, there exists a constant $c>0$, depending only on $\mathbb {E}$, such that for all $H\ge 1$ and all $A\in \mathfrak {o}^{d+1}$ holds

18 $$\begin{aligned} \vert N_{K/\mathbb {Q}}({\text {disc}}(f_A)) \vert \le c H^{(2d-2)k}, \end{aligned}$$

where $k=[K:\mathbb {Q}]$. Thus, combining (17) and (18) and taking the logarithm, we obtain for $H\ge 1$

$$\begin{aligned} \alpha \vert \{ \mathfrak {p}\in \mathcal {P}_K \ \mid \ \mathfrak {p}\succeq H^\alpha , A \in U_\mathfrak {p} \} \vert \le \ln (c) + (2d-2) k. \end{aligned}$$

Hence, Condition (5) holds for any choice of $\alpha >0$.

Now we check Conditions (6) and (7). Let $\mathbb {E}$ be an integral basis of $\mathfrak {o}$, $k=[K:\mathbb {Q}]$ and $\varphi : \mathfrak {o}\rightarrow \mathbb {Z}^k$ be the isomorphism of $\mathbb {Z}$-modules induced by the basis $\mathbb {E}$. We get

$$\begin{aligned} \frac{ \left| \bigcap _{j=1}^n U_{\mathfrak {p}_j} \cap O(H, \mathbb {E})^{d+1} \right| }{ (2H)^{(d+1)k}} \le \left( \frac{ \left| \bigcap _{j=1}^n \mathfrak {p}_j \cap O(H, \mathbb {E}) \right| }{(2H)^k} \right) ^d. \end{aligned}$$

Hence, we are only interested in ideals of the form $\bigcap _{j=1}^n \mathfrak {p}_j$. In fact, as $\varphi$ preserves densities of lattices, it is enough to consider its image in $\mathbb {Z}^k$ under $\varphi$. By a similar argument as for [1, Proposition 1] one can show that for any $n\in \mathbb {N}$ there exists a universal constant $c>0$ such that for any lattice $\Gamma \subseteq \mathbb {R}^n$ of full rank and all $H\in \mathbb {N}_{\ge 1}$ holds

$$\begin{aligned} \left| \frac{\vert \Gamma \cap [-H, H)^n\vert }{(2H)^n} - \rho (\Gamma ) \right| \le c \rho (\Gamma ) \left( \frac{{\text {diam}}(\Gamma )}{H} + \left( \frac{{\text {diam}}(\Gamma )}{H} \right) ^2 \right) , \end{aligned}$$

where ${\text {diam}}(\Gamma )$ denotes the diameter of the fundamental domain of $\Gamma$.

Hence, if ${\text {diam}}( \varphi ( \bigcap _{j=1}^n \mathfrak {p}_j)) \le cH$, then we can estimate

19 $$\begin{aligned} \begin{aligned}&\frac{\left| \bigcap _{j=1}^n \mathfrak {p}_j \cap O(H, \mathbb {E}) \right| }{(2H)^k} = \frac{\left| \varphi \left( \bigcap _{j=1}^n \mathfrak {p}_j\right) \cap [-H,H)^k \right| }{(2H)^k} \le c' \rho \left( \varphi \left( \bigcap _{j=1}^n \mathfrak {p}_j \right) \right) \\&\quad = c' \rho \left( \bigcap _{j=1}^n \mathfrak {p}_j \right) = c' \prod _{j=1}^n [\mathfrak {o}: \mathfrak {p}_j]^{-1} = c' \prod _{j=1}^n p_j^{-{\text {deg}}(\mathfrak {p}_j)} \le c' \prod _{j=1}^n \frac{1}{p_j}. \end{aligned} \end{aligned}$$

Thus, for $d\ge 2$ we can pick $v_\mathfrak {p} = (1+c')^d/p^d$ and the series converges. All we need to show is that there exists some $\alpha >0$ and some universal constant $C>0$ such that for $\mathfrak {p}_1, \dots , \mathfrak {p}_n \preceq H^\alpha$ holds

$$\begin{aligned} {\text {diam}}\left( \varphi \left( \bigcap _{j=1}^n \mathfrak {p}_j \right) \right) \le C H. \end{aligned}$$

However, by Minkowski’s Second Theorem [5, Chapter VIII.2, Theorem 1] we have for any ideal $\mathfrak {a} \in \mathfrak {o}$

$$\begin{aligned} {\text {diam}}(\varphi (\mathfrak {a}))\le \rho (\varphi (\mathfrak {a}))^{-1} = \rho (\mathfrak {a})^{-1} = [\mathfrak {o}: \mathfrak {a}]. \end{aligned}$$

By the Chinese Remainder Theorem we get

$$\begin{aligned} {\text {diam}}\left( \varphi \left( \bigcap _{j=1}^n \mathfrak {p}_j\right) \right) \le [\mathfrak {o}: \bigcap _{j=1}^n \mathfrak {p}_j] = \prod _{j=1}^n [\mathfrak {o}: \mathfrak {p}_j] \le \prod _{j=1}^n p_j^k, \end{aligned}$$

Hence, if $\mathfrak {p}_1, \dots , \mathfrak {p}_n \preceq H^\alpha$, then we get

$$\begin{aligned} {\text {diam}}\left( \bigcap _{j=1}^n \mathfrak {p}_j \right) \le H^{\alpha n k}. \end{aligned}$$

Choosing $\alpha = 1/(nk)$, we obtain

$$\begin{aligned} {\text {diam}}\left( \bigcap _{j=1}^n \mathfrak {p}_j \right) \le H. \end{aligned}$$

$$\square $$

The argument of Corollary 4.8 can be generalized to shifted Eisenstein polynomials as well.

#### Corollary 4.9

Let $d\ge 3$ be an integer and let *K* be number field and $\widetilde{\Gamma }^d$ be the shifted Eisenstein polynomials of degree *d*. We associate the system

$$\begin{aligned} \widetilde{U}_\mathfrak {p}= \bigcup _{b+\mathfrak {p}\in \mathfrak {o}/\mathfrak {p}}\sigma _b^{-1}\left( (\mathfrak {p}\mathfrak {o}_\mathfrak {p}\setminus \mathfrak {p}^2 \mathfrak {o}_\mathfrak {p}) \times (\mathfrak {p} \mathfrak {o}_\mathfrak {p})^{d-1} \times (\mathfrak {o}_\mathfrak {p}\setminus \mathfrak {p} \mathfrak {o}_\mathfrak {p})\right) \end{aligned}$$

and $\widetilde{U}_\infty = \emptyset$. Then the system $(\widetilde{U}_\mathfrak {p})_{\mathfrak {p}\in N_K}$ satisfies the conditions of Theorem 3.5 for any $n\in \mathbb {N}$.

In particular, we have

$$\begin{aligned} \rho (\widetilde{\Gamma }^d)&= \left( 1-\prod \limits _{\mathfrak {p}\in \mathcal {P}_K}\left( 1-\frac{(N(\mathfrak {p})-1)^2}{N(\mathfrak {p})^{d+1}}\right) \right) , \\ \mu&= \sum \limits _{\mathfrak {p}\in \mathcal {P}_K}\frac{(N(\mathfrak {p})-1)^2}{N(\mathfrak {p})^{d+1}}, \quad \mu _{\widetilde{\Gamma }^d} = \rho (\widetilde{\Gamma }^d)^{-1} \mu , \end{aligned}$$

where $$N(\mathfrak {p})= \vert \mathfrak {o}/\mathfrak {p} \vert = p^{\deg (\mathfrak {p})}$$ and $$\deg (\mathfrak {p}) = [ \mathfrak {o}/ \mathfrak {p}: \mathbb {F}_p]$$.

Furthermore, the restricted variance is given by

$$\begin{aligned} \sigma ^2_{\widetilde{\Gamma }^d} = \frac{1}{\rho (\widetilde{\Gamma }^d)}\left( \mu ^2 - \sum \limits _{\eta \in \mathcal {P}_K} \left( \frac{(N(\mathfrak {p})-1)^2}{N(\mathfrak {p})^{d+1}}\right) ^2 + \mu \right) - \frac{2\mu _{\widetilde{\Gamma }^d}}{\rho (\widetilde{\Gamma }^d)} \mu + \mu _{\widetilde{\Gamma }^d}^2. \end{aligned}$$

#### Proof

Recall that we already have verified (4) in the proof of Corollary 4.7.

To show (5) we just note that the discriminant of *f*(*x*) is equal to the discriminant of $f(x+a)$ for any $a\in \mathfrak {o}$. Hence, the same proof as in Corollary 4.8 works to verify (5) for any $\alpha >0$.

In order to verify (6) and (7), we use Theorem 4.6 to obtain

$$\begin{aligned} \bigcap _{j=1}^n \widetilde{U}_{\mathfrak {p}_j} \cap O(H, \mathbb {E})^{d+1}&= \bigcap _{j=1}^n \widetilde{\Gamma }_{\mathfrak {p}_j}^d \cap O(H, \mathbb {E})^{d+1} \\&= \bigcap _{j=1}^n \bigsqcup _{b_j + \mathfrak {p}_j \in \mathfrak {o}/ \mathfrak {p}_j} \sigma _{b_j}^{-1} (\Gamma _{\mathfrak {p}_j}^d) \cap O(H, \mathbb {E})^{d+1} \\&= \bigcup _{b_1+\mathfrak {p}_1\in \mathfrak {o}/\mathfrak {p}_1, \dots , b_n+\mathfrak {p}_n \in \mathfrak {o}/\mathfrak {p}_n} \bigcap _{j=1}^n \sigma _{b_j}^{-1}(\Gamma _{\mathfrak {p}_j}^d) \cap O(H, \mathbb {E})^{d+1}. \end{aligned}$$

By the Chinese Remainder Theorem there exists $b\in \mathfrak {o}$ such that $b+ \mathfrak {p}_j = b_j + \mathfrak {p}_j$ for all $j\in \{1, \dots , n\}$ and thus, by Proposition 4.4, we have $\sigma _{b_j}^{-1}(\Gamma _{\mathfrak {p}_j}^d) = \sigma _{b}^{-1}( \Gamma _{\mathfrak {p}_j}^d)$. This implies

$$\begin{aligned} \bigcap _{j=1}^n \sigma _{b_j}^{-1} (\Gamma _{\mathfrak {p}_j}^d) = \bigcap _{j=1}^n \sigma _b^{-1} (\Gamma _{\mathfrak {p}_j}^d) = \sigma _b^{-1} \left( \bigcap _{j=1}^n \Gamma _{\mathfrak {p}_j}^d \right) . \end{aligned}$$

Combining the previous identities yields

$$\begin{aligned} \bigcap _{j=1}^n \widetilde{U}_{\mathfrak {p}_j} \cap O(H, \mathbb {E})^{d+1} = \bigcup _{b + \bigcap _{j=1}^n \mathfrak {p}_j \in \mathfrak {o}/ \bigcap _{j=1}^n \mathfrak {p}_j} \sigma _b^{-1} \left( \bigcap _{j=1}^n \Gamma _{\mathfrak {p}_j}^{d+1} \right) \cap O(H, \mathbb {E})^{d+1}. \end{aligned}$$

Hence, we choose $\alpha = 1/(nk)$ and denote by $\varphi : \mathfrak {o}\rightarrow \mathbb {Z}^k$ again the isomorphism induced by $\mathbb {E}$. By the same argument as in the proof of Corollary 4.8 we get that for $\mathfrak {p}_1, \dots , \mathfrak {p}_n \preceq H^\alpha$ we have ${\text {diam}}(\varphi (\bigcap _{j=1}^n \mathfrak {p}_j)) \le H$ and thus by the computation (19) we obtain

$$\begin{aligned} \frac{\left| \bigcap _{j=1}^n \widetilde{U}_{\mathfrak {p}_j} \cap O(H, \mathbb {E})^{d+1} \right| }{(2H)^{k(d+1)}}&\le \left( \prod _{j=1}^n p_j^{{\text {deg}}(\mathfrak {p}_j)} \right) \frac{\left| \bigcap _{j=1}^n U_{\mathfrak {p}_j} \cap O(H, \mathbb {E})^{d+1} \right| }{(2H)^{k(d+1)}} \\&\le \prod _{j=1}^n p_j^{-(d-1){\text {deg}}(\mathfrak {p}_j)} \le \prod _{j=1}^n \frac{1}{p_j^2}, \end{aligned}$$

where we used $d\ge 3$ to get the last inequality. Therefore, also (6) and (7) are verified. Thus, all the claims follow from Theorem 3.5. $\square$

#### Remark 4.10

Note that Corollary 4.9 does not extend to $d=2$. For $d=2$ the conditions of Theorem 3.5 are not satisfied for any positive integer. In fact, the system does not even satisfy the weaker conditions of Theorem 2.4 as $\sum _{\eta \in N_K} s_\eta$ diverges, as we saw in the proof of Corollary 4.7.

**Supplementary information** Not applicable.

## Declarations

### Data availability

Data sharing not applicable to this article as no datasets were generated or analysed during the current study.
